# PREVALENCE AND RISK FACTORS FOR ADRD AMONG ARAB AMERICANS

**DOI:** 10.1093/geroni/igz038.1997

**Published:** 2019-11-08

**Authors:** Florence Dallo

**Affiliations:** Oakland University, Rochester, Michigan, United States

## Abstract

In the United States (U.S.), Alzheimer’s Disease and Related Dementias (ADRD) afflict over 4.7 million individuals ages 65 or older. Arab Americans are a subgroup of whites in which ADRD is not well understood. This study estimates prevalence and risk factors for ADRD among Arab Americans ages 45 or older. Data for 2000-2017 from the National Health Interview Survey (NHIS) using the region of birth question was used (N=222,219). The age- and sex-adjusted prevalence of ADRD was 10.3% for foreign-born Arab Americans compared to approximately 7.5% for US-born non-Hispanic whites (NHW), blacks and Asians. The prevalence of ADRD was 8.6% for Hispanics (all p-values <.0001). When controlling for age and sex, Arab Americans were 1.4 times (OR=1.02,1.93) more likely to have ADRD compared to US-born NHW. Future studies should capture other generations of Arab Americans to better understand the trend of ADRD among this understudied, often invisible population.

